# Exploring *GBA1* gene in Parkinson's disease: Prevalence and variant spectrum from Asia minor

**DOI:** 10.1007/s10072-025-08286-5

**Published:** 2025-06-20

**Authors:** Merve Koç Yekedüz, Rezzak Yilmaz, Talha Abali, Sema Nur Kibrit, Ahmet Veli Karacan, Elif Yüsra Unutmaz, Gülnur Ayık, Dudu Genç-Batmaz, G. Rana Dilek, Binnur Çelik, Emine Gemci, Turgut Şahin, Ahmet Yalcin, Serdar Ceylaner, M. Cenk Akbostancı, Fatma Tuba Eminoğlu

**Affiliations:** 1 Department of Pediatric Metabolism, Ankara University School of Medicine, Ankara, Turkey; 2 Department of Anesthesiology, Harvard Medical School, Boston Children’s Hospital, Critical Care and Pain Medicine, Boston, MA USA; 3 Ankara University Brain Research Center, Ankara, Turkey; 4 Department of Neurology, Ankara University School of Medicine, Ankara, Turkey; 5 Ankara University School of Medicine, Ankara, Turkey; 6 Department of Geriatrics, Ankara University School of Medicine, Ankara, Turkey; 7 Intergen Genetic and Rare Diseases Diagnosis and Research Center, Ankara, Turkey; 8 Lokman Hekim University, Medical Faculty, Department of Medical Genetics, Ankara, Turkey; 9 Ankara University Rare Diseases Application and Research Center, Ankara, Turkey

**Keywords:** Beta-glucocerebrosidase, *GBA1*, Parkinson's disease, Whole exome sequencing

## Abstract

**Background:**

The *GBA1* gene has been established as a notable risk factor in Parkinson's disease (PD). While some population-specific variants were reported, many regions of the world remain underexplored. This study investigates the prevalence, types, and clinical associations of *GBA1* variants in a large cohort of patients with PD (PwP) from Turkey.

**Methods:**

A total of 716 individuals, including 513 PwP and 203 healthy controls (HC), were evaluated. Genetic analysis of *GBA1* variants was performed using nextgeneration sequencing. Additionally, whole exome sequencing (WES) was conducted on participants with detected *GBA1* variants. Clinical data, including motor, non-motor, and quality of life assessments, were collected. Enzyme and substrate levels were measured from dry blood spot samples.

**Results:**

*GBA1* variants were found in 13.2% of PD patients, significantly higher than in HC (6.4%), corresponding to an average 2.2-fold higher prevalence. The most frequent variants were p.T369M, p.L444P, and p.N370S. Additionally, 15 variants not previously reported in PD were detected. Patients with pathogenic variants had an earlier age of onset including a higher levodopa-equivalent daily dose and motor complications.

Enzyme and substrate levels did not differ significantly between the groups. In one patient, WES data showed a *CTSB* variant which was reported to modify the effects of *GBA1*.

**Conclusion:**

This is the largest study revealing prevalence of *GBA1* variants among PwP in Turkey, with significant clinical implications. The findings enrich the literature by expanding the previously unknown landscape of *GBA1* variants in this region.

**Supplementary Information:**

The online version contains supplementary material available at 10.1007/s10072-025-08286-5.

## Introduction

Genetics has been increasingly recognized in the clinical routine and research in Parkinson’s disease (PD) [1–3]. Moreover, genetic influences in PD are no longer limited to monogenic forms, comprising of a list of genes directly related to PD [4]. Today, they also include evaluating potential variants associated with PD, despite not being directly causative [5]. The complex interplay between sets of genes offers insights into various aspects of PD, including pathophysiological mechanisms, gene-environment interactions, risk prediction, and drug development.

The most important genetic risk factor for PD is associated with the *GBA1* gene, which encodes the lysosomal glucosylceramidase (glucocerebrosidase, GCase) enzyme. GCase functions by removing glucose from glucosylceramide and glucosphingosine, as well as contributing to the lysosomal degradation of alpha-synuclein (a-syn) [6]. Disruption in the GCase function due to pathogenic *GBA1* variants leads to an accumulation of a-syn, which further impairs GCase activity, creating a vicious cycle known as the bi-directional loop [7]. The literature suggests that pathological *GBA1* variants are linked to a 2 to 20-fold increased risk for PD in healthy individuals [8], and to an earlier diagnosis and faster progression in the course of PD [9, 10].

Identifying the association between the *GBA1* gene and PD [11] prompted the investigation of the lysosomal mechanisms in PD. Over time, these efforts showed promise for a long-awaited clue to understanding the subtypes of PD and altering the course of the synucleinopathy-related neurodegeneration [12–14]. However, existing data on PD genetics were primarily derived from West European and North American populations, leading to a bias against underrepresented populations and hindering the generalizability of outcomes, which has been underscored as a crucial unmet need [1, 15]. In line with that, while prevalence and variant types of *GBA1*-PD have been documented in various populations, comprehensive data from Asia Minor remains lacking. In a preliminary analysis, data on a selected group of Turkish patients with PD (PwP) were presented [16]. In the current study, we attempted to explore the prevalence, types, and clinical associations of *GBA1* variants in a large cohort of PwP.

### Methods

Between September 2020 and July 2023, PwP and healthy controls (HC) were evaluated at the Ankara University School of Medicine, Departments of Neurology, and Geriatric Medicine. Extensive clinical data, including demographics, comorbidities, motor, non-motor, and quality of life assessments, were collected from PwP.

The whole exome sequencing (WES) was conducted in PwP with variants detected in the *GBA1* gene. Variants were classified according to ACMG criteria and the *GBA1*-PD browser [17]. Population frequencies were stated according to gnomAD (Aggregated) and Turkish Variome by using the website of Franklin by Genoox website (https://franklin.genoox.com/clinical-db/home). Variant confirmation studies were done with targeted sequencing performed by next-generation sequencing using Miseq-Illumina equipment (Illumina, San Diego, CA, USA). *GBA1* gene is amplified in 3 amplicons, the primer sequences are: for exon 1 to 4 TTCCTAAAGTTGTCACCCATACATGC and CCGACAGAATGGGCAGAGTGAGAT, for exon 5 to 7 TTGGTTCCTGTTTTAATGCCCTGTG and CCTAGAAAGGTTTCAAGCGACAACTG and for exon 8 to 11 ATTCTTCCCGTCACCCAMCTCCAG and GTAAGCTCACACTGGCCCTGCTG. For PCR amplification Mytaq DNA polymerase (Meridian Bioscience) enzyme is used according to the manufacturer's recommendations. Thermal cycler protocol used is: 950 C for 5 min; 40 cycles of 950 C for 20 s., 600 C for 20 s., 720 C for 40 s.; 720 C for 5 min.; store at 40C. Beta-glucocerebrosidase and Lyso-Gb1 levels were quantified from dry blood spot (dBS) samples obtained from participants carrying variants in the *GBA1*. To prepare the dBS samples, 60 µL of whole blood was applied onto a Guthrie filter card, followed by air drying at room temperature (3–5 h). The dried samples were subsequently stored at −20 °C for 48 h prior to analysis. Before the biochemical assessment, 3.2 mm diameter discs were carefully punched from the dBS samples. For each participant, these discs were placed in tubes containing an extraction solution and an internal standard solution to facilitate analysis. The samples underwent incubation at 37 °C for 17 h, followed by centrifugation to separate the supernatant for further examination. Beta-glucocerebrosidase enzymatic activity was determined using the 4-methylumbelliferone (4-MU) fluorimetric assay, while Lyso-Gb1 quantification was performed via liquid chromatography-mass spectrometry (LC/MS). For beta-glucocerebrosidase activity, values > 1.30 nmol/mL/hour were considered normal. For Lyso-Gb1, the reference interval was 0.00—14.00 ng/mL. The analytical methodology was conducted in accordance with previously established protocols [18, 19]. Approval of the study was granted by the Ethics Committee of Ankara University School of Medicine, and all procedures were in accordance with the Declaration of Helsinki. Study data were collected and managed using REDCap electronic data capture tools hosted at Ankara University Department of Neurology [20]. Details of the clinical, biochemical and genetic assessments are given in the supplement.

### Statistics

Analyses were performed with an explorative approach. First, the prevalence of all *GBA1* variants was compared between PwP and HC. Then PwP were divided into two groups. PwP who did not carry a *GBA1* variant were assigned to PwP-wild type for *GBA1* (PwP-WT). PwP carrying pathogenic, likely pathogenic, severe, mild, or risk variants for either Gaucher disease (GD) or PD were grouped as PwP-pathogenic impact *GBA1* (PwP-pat*GBA1*). These groups were compared with regard to their demographics, clinical evaluations, and serum enzyme/substrate levels using Chi-square, Student’s t-test, or Mann–Whitney U test as appropriate. Detected features were tested using regression models with appropriate confounders. PwP carrying VUS, unknown, benign, or likely benign variants for both GD and PD (non-pathogenic variants, PwP-non-pat*GBA1*) were excluded from these comparisons. In addition, PwP-patGBA1 and PwP-non-patGBA1 groups were compared in terms of enzyme/substrate levels*.* SPSS Statistics 22.0.0 (SPSS Ltd., Chicago IL) was used for statistical analyses. P-value < 0.05 was considered significant, and no correction was applied for multiple testing.

## Results

### Prevalence and variant types of GBA1

**Fig. 1:**
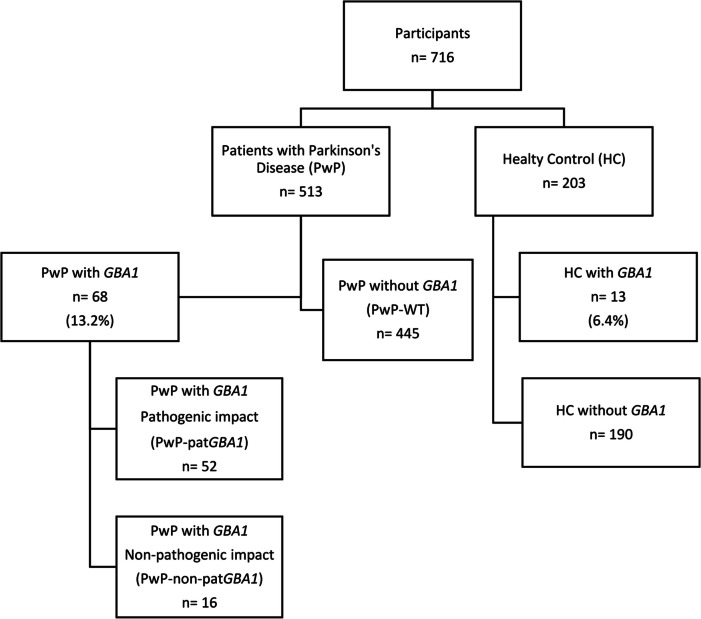
The flowchart of the participants

Our study included 716 individuals, comprising 513 PwP and 203 HC (Fig. 1). Within the PwP cohort, the presence of a *GBA1* variant was observed in 13.2% (n = 68), which was significantly higher compared to HC (n = 13, 6.4%, Chi-square test, p = 0.009). Thus, the odds of having a *GBA1* variant in the PwP group were 2.2 times higher compared to the HC group. Moreover, among the patients harboring a *GBA1* variant, 52 (76.4%) exhibited variants with recognized pathogenic consequences for GD or PD (PD-pat).

The most prevalent heterozygous variants observed in the PwP group were p.T369M (17.6%, n = 12), p.L444P (11.8%, n = 8), and p.N370S (10.3%, n = 7). Fifteen GBA1 variants not previously reported in PwP were identified in our study (Table 1). Based on ACMG criteria and in‑silico predictions, seven of these were classified as variants of uncertain significance (VUS) and eight as likely pathogenic (Supplementary Table 7). In addition, two patients had compound heterozygous variants (p.P67L + p.L444P, p.T369T + p.D409H), and four had homozygous variants (two with IVS6-18 T > A, one with p.Q182Q and one with p.T369M). Among the four PwP and two HC with biallelic variants (Table 1), none exhibited clinical manifestations indicative of GD.

**Table 1:** Detected *GBA1* variants in 513 Turkish PwP and 203 HC

N (%)	Gene	Exon	HGVS.p	HGVS.c	HGVS.p—full length name	rsID	Genotype	Reported in GD	Impact for GD	Reported in PD	Impact for PD	Population Frequencies 1) gnomAD 2) Turkish Variome
Patients with *GBA1* variants												
Patients with *GBA1*-heterozygous												
12 (17.6)	*GBA1*	8	p.T369M	c.1223C > T	p.(Thr408Met)	rs75548401	Het	No	Benign	Yes	RV	1) VRVar (0.61%) 2) VRVar (0.69%)
8 (11.8)	*GBA1*	10	p.L444P	c.1448 T > C	p.(Leu483Pro)	rs421016	Het	Yes	Pat	Yes	Severe	1) VRVar (0.13%) 2) N/A
7 (10.3)	*GBA1*	9	p.N370S	c.1226A > G	p.(Asn409Ser)	rs76763715	Het	Yes	Pat	Yes	Mild	1) VRVar (0.22%) 2) VRVar (0.18%)
4 (5.9)	*GBA1*	8	p.T369T	c.1224G > A	p.(Thr408Thr)	rs138498426	Het	No	VUS	Yes	Unknown	1) VRVar (0.03%) 2) VRVar (0.39%)
3 (4.4)	*GBA1*	7	IVS6-18 T > A	c.762-18 T > A	-	rs140335079	Het	No	Benign	Yes	RV	1) VRVar (0.75%) 2) VRVar 0.60%
2 (2.9)	*GBA1*	8	p.E326K	c.1093G > A	p.(Glu365Lys)	rs2230288	Het	No	VUS	Yes	RV	1) RVar (1.07%) 2) VRVar (0.24%)
2 (2.9)	*GBA1*	7	p.H255Q	c.882 T > G	p.(His294Gln)	rs367968666	Het	Yes	LP	Yes	Severe	1) VRVar (0.02%) 2) VRVar (0.11%)
2 (2.9)	*GBA1*	11	IVS10-10_9delTGinsGA	c.1506-10_9delTGinsGA	-	-	Het	No	VUS	No	Unknown	1) N/A 2) N/A
2 (2.9)	*GBA1*	6	p.G202R	c.721G > A	p.(Gly241Arg)	rs409652	Het	Yes	Pat	Yes	Severe	1) VRVar (0.003%) 2) VRVar (0.02%)
1 (1.5)	*GBA1*	1	IVS1 + 2 T > G	c.27 + 2 T > G	-	-	Het	Yes	VUS	Yes	Severe	1) N/A 2) N/A
1 (1.5)	*GBA1*	10	p.A446A	c.1455A > G	p.(Ala485Ala)	rs199928507	Het	No	VUS	Yes	Unknown	1) VRVar (0.01%) 2) VRVar (0.08%)
1 (1.5)	*GBA1*	10	p.A456P	c.1483G > C	p.(Ala495Pro)	rs368060	Het	Yes	Pat	Yes	Unknown	1) VRVar (0.01%) 2) N/A
1 (1.5)	*GBA1*	10	IVS9-2_5dupTTCA	c.1389-1_1389insTTCA	-	-	Het	No	LP	No	Unknown	1) N/A 2) N/A
1 (1.5)	*GBA1*	6	p.A229V	c.686C > T	p.(Ala229Val)	rs75636769	Het	No	LP	No	Unknown	1) VRVar (0.002%) 2) N/A
1 (1.5)	*GBA1*	9	p.Y457*	c.1371C > A	p.(Tyr457Ter)	-	Het	No	LP	No	Unknown	1) N/A 2) N/A
1 (1.5)	*GBA1*	7	p.L325F	c.975G > C	p.(Leu325Phe)	-	Het	No	VUS	No	Unknown	1) N/A 2) N/A
1 (1.5)	*GBA1*	7	p.L288P	c.863 T > C	p.(Leu288Pro)	-	Het	Yes	LP	No	Unknown	1) N/A 2) N/A
1 (1.5)	*GBA1*	1 (5’UTR)	-	c.−14A > G	-	rs1064640	Het	No	VUS	No	Unknown	1) VRVar (0.001%) 2) N/A
1 (1.5)	*GBA1*	11	p.Y531Y	c.1593C > T	p.(Tyr531Tyr)	-	Het	No	VUS	No	Unknown	1) N/A 2) N/A
1 (1.5)	*GBA1*	6	p.G234R	c.700G > A	p.(Gly234Arg)	-	Het	No	LP	No	Unknown	1) N/A 2) N/A
1 (1.5)	*GBA1*	9	p335fs	c.1265_1319del	p.(Leu422ProfsTer4)	rs80356768	Het	Yes	Pat	Yes	Unknown	1) N/A 2) N/A
1 (1.5)	*GBA1*	5	IVS5 + 17 T > C	c.588 + 17 T > C	-	rs761359412	Het	No	VUS	No	Unknown	1) VRVar (0.001%) 2) N/A
1 (1.5)	*GBA1*	10	p.D492N	c.1474G > A	p.(Asp492Asn)	rs779958429	Het	Yes	LP	Yes	LP	1) VRVar (0.001%) 2) VRVar (0.06%)
1 (1.5)	*GBA1*	7	p.L279M	c.835C > A	p.(Leu279Met)	-	Het	No	VUS	No	Unknown	1) N/A 2) N/A
1 (1.5)	*GBA1*	3	p.Y50C	c.149A > G	p.(Tyr50Cys)	-	Het	No	VUS	No	Unknown	1) N/A 2) N/A
1 (1.5)	*GBA1*	3	p.K27R	c.38A > G	p.(Lys13Arg)	rs150466109	Het	Yes	Benign	Yes	Benign	1) RVar (0.74%) 2) N/A
1 (1.5)	*GBA1*	2	IVS2 + 1G > A	c.115 + 1G > A	-	rs104886460	Het	Yes	Pat	Yes	Severe	1) VRVar (0.01%) 2) N/A
1 (1.5)	*GBA1*	5	p.N156Hfs*5	c.465_481del	p.(Asn156fs)	-	Het	No	LP	No	Unknown	1) N/A 2) N/A
1 (1.5)	*GBA1*	9	p.R434P	c.1301G > C	p.Arg434Pro	-	Het	Yes	LP	No	Unknown	1) N/A 2) N/A
Patients with GBA1-compound heterozygous												
V1a	*GBA1*	3	p.P67L	c.200C > T	p.(Pro67Leu)	-	Het	No	LP	No	Unknown	1) N/A 2) N/A
V2a	*GBA1*	10	p.L444P	c.1448 T > C	p.(Leu483Pro)	rs421016	Het	Yes	Pat	Yes	Severe	1) VRVar (0.13%) 2) N/A
V1b	*GBA1*	8	p.T369T	c.1224G > A	p.(Thr408Thr)	rs138498426	Het	No	VUS	Yes	Unknown	1) VRVar 0.03% 2) VRVar 0.39%
V2b	*GBA1*	9	p.D409H	c.1342G > C	p.(Asp448His)	rs1064651	Het	Yes	Pat	Yes	Severe	1) VRVar (0.001%) 2) N/A
Patients with GBA1-homozygous												
2 (2.9)	*GBA1*	7	IVS6-18 T > A	c.762-18 T > A	-	rs140335079	Homo	No	Benign	Yes	RV	1) VRVar (0.75%) 2) VRVar (0.60%)
1 (1.5)	GBA1	5	p.Q182Q	c.546G > A	p.(Gln182Gln)	rs545391048	Homo	Yes	Likely Benign	Yes	Unknown	1) VRVar (0.037%) 2) VRVar (0.13%)
1 (1.5)	*GBA1*	8	p.T369M	c.1223C > T	p.(Thr408Met)	rs75548401	Homo	No	Benign	Yes	RV	1) VRVar (0.61%) 2) VRVar (0.69%)
Healthy controls with *GBA1* variants												
Healthy controls with *GBA1*- heterozygous												
1	*GBA1*	8	p.N372N	c.1116C > T	p.(Asn372Asn)	rs778140625	Het	No	VUS	No	Unknown	1) VRVar (0.004%) 2) N/A
1	*GBA1*	2	IVS2 + 1G > A	c.115 + 1G > A	-	rs104886460	Het	Yes	Pat	Yes	Severe	1) VRVar (0.01%) 2) N/A
1	*GBA1*	7	p.H255Q	c.882 T > G	p.(His294Gln)	rs367968666	Het	Yes	LP	Yes	Severe	1) VRVar (0.02%) 2) VRVar (0.11%)
1	*GBA1*	3	p.R41H	c.122G > A	p.(Arg41His)	rs751095441	Het	No	VUS	No	Unknown	1) VRVar (0.001%) 2) VRVar (0.04%)
1	*GBA1*	10	p.L444P	c.1448 T > C	p.(Leu483Pro)	rs421016	Het	Yes	Pat	Yes	Severe	1) VRVar (0.13%) 2) N/A
1	*GBA1*	5	p.I158I	c.474C > T	p.(Ile158Ile)	rs147411159	Homo	No	Likely benign	Yes	Unknown	1) VRVar (0.07%) 2) N/A
1	*GBA1*	8	p.T369M	c.1223C > T	p.(Thr408Met)	rs75548401	Het	No	Benign	Yes	RV	1) VRVar (0.61%) 2) VRVar (0.69%)
1	*GBA1*	7	IVS6-18 T > A	c.762-18 T > A	-	rs140335079	Het	No	Benign	Yes	Unknown	1) VRVar (0.75%) 2) VRVar (0.60%)
1	*GBA1*	9	p.N370S	c.1226A > G	p.(Asn409Ser)	rs76763715	Het	Yes	Pat	Yes	Mild	1) VRVar (0.22%) 2) VRVar (0.18%)
Healthy controls with -compound heterozygous												
V1	*GBA1*	9	p.N370S	c.1226A > G	p.(Asn409Ser)	rs76763715	Het	Yes	Pat	Yes	Mild	1) VRVar (0.22%) 2) VRVar (0.18%)
V2	*GBA1*	10	p.V460M	c.1495G > A	p.(Val499Met)	rs369068553	Het	No	LP	Yes	Unknown	1) VRVar (0.02%) 2) N/A
V1	*GBA1*	10	p.A456P	c.1483G > C	p.(Ala495Pro)	rs368060	Het	Yes	Pat	Yes	Unknown	1) VRVar (0.01%) 2) N/A
V2	*GBA1*	10	p.L444P	c.1448 T > C	p.(Leu483Pro)	rs421016	Het	Yes	Pat	Yes	Severe	1) VRVar (0.13%) 2) N/A
Healthy controls with *GBA1*- homozygous												
1	*GBA1*	7	IVS6-18 T > A	c.762-18 T > A	-	rs140335079	Homo	No	Benign	Yes	Unknown	1) VRVar (0.75%) 2) VRVar (0.60%)
1	*GBA1*	8	p.T369M	c.1223C > T	p.(Thr408Met)	rs75548401	Homo	No	Benign	Yes	RV	1) VRVar (0.61%) 2) VRVar (0.69%)

HGVS, Human Genome Variation Society; GD, Gaucher disease; Het, heterozygous; Homo, homozygous; Pat, pathogenic; PD, Parkinson’s disease; RV, risk variant; VUS: variant of unknown significance, LP, likely pathogenic

### Enzyme and substrate levels

No significant differences were observed between PwP and HC groups with a detected *GBA1* variant regarding beta-glucocerebrosidase and Lyso-Gb1 levels (Table 2). Likewise, PwP-pat*GBA1* and PwP-non-pat*GBA1* groups did not significantly differ in terms of beta-glucocerebrosidase and Lyso-Gb1 levels (p = 0.116, p = 0.265; respectively) (Table 3). In participants with biallelic variants (four PwP, two HC), the levels of beta-glucocerebrosidase and Lyso-Gb1 were within normal ranges.

**Table 2:** Prevalence of *GBA1* variants and enzymatic activity in PwP and HC

	PwP (n = 513)	HC (n = 203)	P-value
Age, years, median (IQR)	66.0 (13.0)	66.0 (30.0)	0.062
Male sex, n (%)	265 (51.7)	106 (52.2)	0.970
*GBA1* variants and enzymatic activity			
Presence of a *GBA1* variant, n (%)	68 (13.2)	13 (6.4)	0.009
Beta-glucoserebrosidase nmol/ml/h, median, (IQR)	3.0 (1.4)	2.9 (1.2)	0.735
Lyso-Gb1, ng/ml, median (IQR)	2.3 (1.9)	2.6 (1.7)	0.936

SD, standard deviation; *GBA1*, glucocerebrosidase; Lyso-Gb1, glucosylsphingosine

ǂ Enzymatic activity was evaluated only in participants with a detected variant

**Table 3:** Enzyme and substrate levels of PwP carrying pathogenic and non-pathogenic variants of *GBA1*

	PwP-pat*GBA1* (n = 52)	PwP-non-pat*GBA1* (n = 16)	P-value
Beta-glucoserebrosidase nmol/ml/h, median, (IQR)*	2.9 (1.1) *	3.5 (2.0)^t^	0.116
Lyso-Gb1, ng/ml, median (IQR)**	2.3 (2.3) **	2.1 (1.2)^tt^	0.265

^*^n = 44, **n = 46, ^t^n = 14, ^tt^n = 14

### Clinical parameters

Extensive clinical data of the PwP were given in Table 4. The results showed that despite similar age range, PwP-pat*GBA1* had a 3.4 years earlier age of onset and thus longer disease duration. In addition, treatment with subthalamic nucleus deep brain stimulation (STN-DBS) was more frequent in PwP-pat*GBA1* compared to PwP-WT (17.6% vs. 6.9%, p = 0.009). Levodopa-equivalent daily dose (LEDD) and MDS-UPDRS-IV scores were also significantly higher in PwP-pat*GBA1* compared to PwP-WT (p = 0.001, p = 0.017, respectively). Most scores in other motor, non-motor assessments, and quality of life parameters were worse in the PwP-pat*GBA1* group but did not reach statistical significance (Table 4). Given that the detected significant effects can be confounded by disease duration, regression models were set by including disease duration as a confounding variable. The logistic regression model for the predicting STN-DBS (as the dependent variable) showed a good fit and correctly classified 32% of the cases (χ2(18) = 64.930, p < 0.001). The model confirmed that PwP-pat*GBA1* were around three times more likely to have STN-DBS (OR = 2.89; 95% CI: 1.17–7.13 p = 0.0021), independent of disease duration (OR = 1.2; 95% CI: 1.15–1.30 p < 0.001) or age (OR = 0.95; 95% CI: 0.92–0.97 p = 0.006). Also, linear regressions were run for LEDD and MDS-UPDRS-IV (given as dependent variables in each model). The first model (F(2, 485) = 67.662, p < 0.0001, R2 = 0.21) showed a marginally significant association between the PwP-pat*GBA1* group and high MDS-UPDRS-IV values (β = 1.13; 95% CI: −0.01–2.28 p = 0.053). With regard to the LEDD scores, the model showed that (F(2, 488) = 69.845, p < 0.001, R2 = 0.22), PwP-pat*GBA*1 received on average 156 mg more levodopa (β = 156; 95% CI: 40–273 p = 0.009) compared to PwP-WT. This effect was also independent of disease duration (β = 35; 95% CI: 29–41 p < 0.001).

**Table 4:** Comparison of demographical and clinical features in PwP with and without pathogenic *GBA1* variants

	PD-WT (n = 445)	PD-pat*GBA1*^t^ (n = 52)	p-value
*Demographics & disease-related information*			
Age, years, mean (SD)	64.4 (10.7)	62.4 (10.4)	0.223
Male sex, n (%)	225 (51)	32 (61.5)	0.151
Age of onset, years, mean (SD)	59.0 (11.9)	55.6 (10.7)	0.047*
Disease duration, years, median (IQR)	3.5 (6.2)	6.0 (7.9)	0.005*
Young-onset PD (≤ 50), n (%)	94 (21.4)	15 (28.8)	0.223
Family history of PD, n (%)^ǂ^	120 (27.6)	17 (32.7)	0.439
History of STN-DBS	27 (6.9)	9 (17.6)	0.009*
Charlson comorbidity index, mean (SD)	3.4 (2.1)	3.0 (1.2)	0.205
*Motor assessments*			
LEDD, mg/day, mean (SD)	597 (447)	829 (500)	0.001*
MDS-UPDRS-II, mean (SD)	14.8 (10.1)	16.3 (9.5)	0.332
MDS-UPDRS-III, “on-state” mean (SD)	33.1 (15.3)	38.3 (19.1)	0.060
MDS-UPDRS-IV, median (IQR)	0 (5)	3 (10)	0.017*
Hoehn & Yahr, mean (SD)	2.2 (1.1)	2.5 (1.1)	0.068
PIGD subtype, n (%)	234 (58.4)	30 (65.2)	0.382
Nine-hole peg test, sec, median (IQR)	30.3 (14.7)	32.2 (17.3)	0.194
Side-to-side tap test, mean (SD)	19.8 (6.1)	18.9 (6.2)	0.339
Timed up and go test, sec, median	12.1 (7.1)	12.5 (7.2)	0.407
*Non-motor assessments*			
MDS-UPDRS-I, mean (SD)	12.5 (7.1)	13.6 (8.5)	0.323
Cognitive impairment, n (%)	241 (55.5)	24 (47.1)	0.250
Hallucinations and psychosis, n (%)	52 (12)	11 (22)	0.053
Depression, n (%)	163 (37.1)	21 (40.4)	0.647
Anxiety, n (%)	152 (34.6)	20 (38.5)	0.583
Apathy, n (%)	163 (37.7)	19 (37.3)	0.947
Dopamine dysregulation syndrome, n (%)	46 (10.5)	6 (11.8)	0.786
Sleep problems, n (%)	264 (61)	33 (64.7)	0.604
Daytime sleepiness, n (%)	314 (72.4)	38 (74.5)	0.744
Pain and other sensations, n (%)	299 (68.4)	34 (66.7)	0.799
Urinary dysfunction, n (%)	298 (68.3)	36 (70.6)	0.744
Constipation, n (%)	227 (51.9)	28 (54.9)	0.689
Orthostatic hypotension, n (%)	147 (33.6)	20 (38.5)	0.488
Fatigue, n (%)	308 (70.6)	33 (64.7)	0.381
Cognitive impairment (based on MMSE scores), n (%)	128 (28.8)	18 (34.6)	0.381
Clock-drawing test, median (IQR)	3 (2)	2 (2)	0.256
*Quality of life*			
PDQ-39 mobility, mean (SD)	16.5 (12.0)	19.5 (11.2)	0.087
PDQ-39 ADL, median (IQR)	6 (11)	8 (11)	0.572
PDQ-39 emotional well-being, mean (SD)	9.5 (5.7)	8.9 (6.3)	0.438
PDQ-39 stigma, median (IQR)	2 (5)	2 (6)	0.759
PDQ-39 social support, median (IQR)	0 (3)	0 (3)	0.883
PDQ-39 cognition, mean (SD)	4.7 (3.3)	5.5 (3.7)	0.122
PDQ-39 communication, median (IQR)	2 (4)	2 (4)	0.873
PDQ-39 bodily discomfort, mean (SD)	4.8 (2.9)	4.1 (3.3)	0.123
PDQ-39 total, mean (SD)	50.5 (28.8)	53.5 (30.0)	0.503

PwP, patients with Parkinson’s disease; SD, standard deviation; IQR, interquartile range; PIGD, postural instability and gait disorder; LEDD, levodopa-equivalent daily dose; *GBA1*, glucocerebrosidase; MMSE, Mini-mental State Examination; PDQ-39, The Parkinson’s Disease Questionnaire; STN-DBS, subthalamic nucleus deep brain stimulation; ADL, activities of daily living. The cut-off value for MMSE to rule out cognitive impairment is ≥ 24

^t^ variants that were reported as VUS/unknown or benign for both GD disease or PD were not included

^*^ p < 0.05

ǂ first and second-degree relatives

### Results of whole exome sequencing (WES)

In this study, WES was performed in those with a detected *GBA1* variant. The data generated by WES were evaluated for monogenic PD, lysosomal disorders, and genetic modifiers of *GBA1* in PD. With respect to variants associated with lysosomal mechanisms, one patient (heterozygous *GBA1:* p. L444P) also harbored a heterozygous variant in *GALC* related to Krabbe disease (c.956A > G chr14-87,965,582 T > C p.Tyr319Cys NM_000153.4). Another female patient with *GBA1*: p.R434P carried a heterozygous variant in *GLA* related to X-linked Fabry disease (c.937G > T chrX-101398432 C > A p.Asp313Tyr NM_000169.3).

Some PwP also had variants listed as monogenic PD. One patient with a disease onset at 64 years with *GBA1:* p.T408T also had a heterozygous variant in the *FBX07* gene (c.555del, p.Leu186fs). Another patient with a heterozygous variant in the *GBA1:* p.G234R, also displayed a heterozygous variant in the *ADH1C* gene (c.232G > T, p.Gly78*). In addition, one patient with a homozygous variant in the *GBA1*:p.T408M showed a deletion in the *PRKN* gene (del chr6:162,443,160–162478459) associated with PD. Another patient with a heterozygous variant in the *GBA1*: p.G241R also had a heterozygous variant in the *VPS35* gene (c.915-2_915-1insT) which is listed as a rare autosomal dominant (AD) monogenic cause of PD. In addition, another patient with heterozygous *GBA1*:0C had a heterozygous variant in the *VPS13C* (c.7761-1G > A), which has been reported to be associated with PD. Finally, one patient with heterozygous *GBA1*:p.L444P with an age of onset of 44 had an additional heterozygous variant in the *DJ-1* gene (c.310G > A, p.Ala104Thr). Clinical data of these patients were given in the supplement.

The WES data were also reviewed for genes that were reported to have a modifying role for the penetrance of *GBA1* variants. One patient with a heterozygous *GBA1*:p.L325F displayed a heterozygous variant in the gene coding cathepsin-B (*CTSB*:c.745G > T, p.Val249Leu), which has a role in lysosomal degradation of a-syn [21]. No variants in the previously reported *GRS* or *SNCA* were detected. Further details of the WES results, including other non-related or potentially related variants detected in PwP-*GBA1* are given in the supplement.

## Discussion

In this study, we attempted to explore the genetic landscape of the *GBA1* variants in PwP in Asia Minor. We found a prevalence of *GBA1* carriage in 13.2% of PwP, with the most common variants being p.T369M, p.L444P, and p.N370S. The impact of these variants on PD has previously been reported as risk, severe, and mild, respectively [22].

The prevalence of the *GBA1* variants in PwP exhibits ethnic differences reported between 2.3–39%. While the frequency ranged 19.2–31.3% in Ashkenazi Jewish PwP, it was much lower in Chinese, ranging 2.4–4.3% [8]. In our study, the prevalence of *GBA1* variants in PwP was similar to the rates reported in Hungarian (15.2%), Dutch (15.0%) [23, 24], Slavic (14.7%) [25] and Italian (14%) [26] populations but higher than those reported in Greek (10.2%) [27], Spanish (9.8%) [28], Polish (8%) [29], Serbian (5.8%) [30], Latin American (5.5%) [31] and Korean (3.2%) [32] studies. Notably, the ROPAD study reported a *GBA1* prevalence of 10.42% among PD patients, which is slightly lower than our findings in the Turkish population [33].

The types of most frequent variants may vary across populations. In our cohort, we report p.T369M (19.1%), p.L444P (13.2%), and p.N370S (10.3%) as the most common variants in Turkey. The most frequent variant in Ashkenazi and non-Ashkenazi Jewish PwP is p.N370S [34, 35]. p.R120W was reported in East Asian studies, and in Europe and West Asia, p.H255Q, p.E326K, or p.N370S have been reported as the most common variants [35]. A recent large-scale review analyzing 27,963 *GBA1* carriers found that among White populations, the most frequent variant was N370S, whereas in Asian and Hispanic populations, L444P was the most common, similar to our cohort [35]. This highlights the substantial variability in *GBA1* variant distribution across different ethnic groups and reinforces the importance of population-specific studies. According to another classification, variants are divided into GD and non-GD [36]. Extensive studies have reported p.L444P and p.N370S as the most common GD-*GBA1* variants and p.E326K and p.T369M as the most common non-GD-*GBA1* variants in PwP [3, 8, 35–37]. In our cohort, we also found p.L444P and p.N370S as the most common GD-*GBA1* variants. Concerning the non-GD-*GBA1* variants, p.T369M and p.T369T were the most common. Despite previous reports that p.T369M is not a risk factor for PD [38–41], a meta-analysis reported otherwise [42]. Similar to the findings from countries such as Netherlands, Belgium, or the UK, and overall White ethnicity, which showed p.E326K or p.T369M as the most frequent non-GD-*GBA1* risk variants [23, 35, 36, 40], we report p.T369M as the most common (19.1%) non-GD-*GBA1* and overall *GBA1* variant in Turkish PwP. In addition, none of our participants carried the non-coding rs3115534-G variant, which has been reported to be present in 39% of PwP with African ancestry [43]. Likewise, the p.D140H, p.E326K and K198E variants, that also seem to have a founder effect for Dutch [23] and Colombian [31] populations, respectively, were non-existent in our cohort.

In this study, we also analyzed the WES data for monogenic PD causes, lysosomal disorders and potential modifier genes that amplify the effect of the *GBA1* gene. Previously, some modifier properties have been identified for *SNCA, CTSB* (encoding cathepsin B), and the lysosomal K^+^ channel *TMEM175* gene [21, 44]. Specifically, risk variants in the *CTSB* locus reduce cathepsin mRNA expression. It has been observed that induced pluripotent neurons from *GBA1* p.N370S mutants have decreased cathepsin B expression compared to controls [45, 46]. In our study, only one male patient carrying a *GBA1* variant had a heterozygous variant in the *CTSB* gene, and the available data are insufficient to discuss it as a modifier. Regarding the lysosomal enzymes, two patients had heterozygous variants in *GALC* (Krabbe disease) and *GLA* (Fabry disease). However, although these enzymes also take part in lysosomal glycosphingolipid degradation pathways, they do work on different substrates and do not directly affect the levels of GCase, and thus, these findings should be regarded as incidental. Considering the monogenic causes, one patient with p.T408T had a heterozygous frameshift mutation (p.Leu186fs) the *FBXO7* gene, which has not been reported in PD. One patient with *GBA1*:p.G234R (unknown for its impact on PD) had a heterozygous variant in the *ADH1C* gene (c.232G > T, p.Gly78*), which has a controversial association with PD [47, 48]. Furthermore, three patients had an additional heterozygous variant in *VPS13C, PRKN,* and *DJ-1* genes, respectively. Given these variants are monoallelic, no additional impact is assumed. However, the *VPS35* variant, which is a very rare form of AD monogenic PD was found in a patient with *GBA1:* p.Y50C which has an unknown impact (Table 1). This 60-year-old female patient had a disease onset of 51 years with mild non-motor burdens and no family history. To our knowledge, the intronic c.915-2_915-1insT variant in *VPS35* has also not been reported earlier [49, 50]. Thus, the effects of both *GBA1*: p.Y50C and the *VPS35* variants on PD in this patient are in question. In contrast to our findings, the co-occurrence of *GBA1* with other monogenic variants was reported mainly with *LRRK2* [33, 35, 37]. The occasional co-occurrence of *GBA1* with *PRKN* and *VPS35* was also reported, but with a much lower frequency than our cohort [33]. Clinical data on co-occurance of *GBA1* with other monogenic variants are given in the supplement.

In our study, the enzyme and substrate level assessments were similar between PwP and controls. GCase enzyme levels have been reported to be slightly lower in PwP compared to the HC group [51]. As expected, PwP carrying biallelic *GBA1* variants also have lower GCase levels compared to heterozygotes [26, 51]. However, neither the enzyme and biomarker values were out of range in PwP with homozygous variants, nor a significant difference was observed between our PwP-pat*GBA1* and PwP-non-pat*GBA1* groups. This is in contrast to a recent large Italian study [26], probably due to our smaller sample size. Regarding the clinical features, we found an earlier disease onset in PwP-pat*GBA1* similar to the previous studies [33, 35]. The PwP-pat*GBA1*1 group also had a higher LEDD, and higher frequency of STN-DBS, and experienced significantly more frequent motor complications. While the the PwP-pat*GBA1* groups had worse scores in other assessments, no significant differences were found (Table 4). The results are in line with the literature suggesting that PwP carrying *GBA1* variants have an earlier disease onset, more severe clinical symptoms and faster progression [9, 10, 52, 53].

A limitation of our study is that enzyme and biomarker levels were not examined in all participants. Biochemical examinations were only conducted in patients with detected *GBA1* variants. Additionally, it should be mentioned that dry blood samples were used in our study. Collecting dry blood samples is inexpensive and easy to transport. However, the results could have been more accurate when studied in leukocytes. Also, the presence of other monogenic causes for PD or other genetic risk factors (such as polygenic risk scores) were not evaluated, and WES was only performed in those with a detected *GBA1* variant. Additionally, this study did not differentiate between levels of cognitive impairment (e.g., mild cognitive impairment or dementia) among patients and education-adjusted norms were not available. Furthermore, the regression model explained only 32% of the variance in DBS eligibility, indicating that additional clinical and genetic variables are required to enhance predictive power. Apart from these limitations, the large sample size, performing WES in PwP with a detected *GBA1* variant, and the detailed clinical evaluations are the strengths of our study. Also, this is the largest sample of PwP from Asia Minor.

In conclusion, we report the prevalence and variant types in a large cohort of Turkish PwP, with 15 variants newly reported in PD. The findings enrich the literature from the previously unknown landscape. More studies are needed to elucidate the impact of novel variants and potential modifiers on *GBA1*.

## Supplementary Information

Below is the link to the electronic supplementary material.

Supplementary file1 (XLSX 80 KB)

Supplementary file2 (DOCX 17 KB)

Supplementary file3 (XLSX 12 KB)

## Data Availability

The data that support the findings of this study are available from the corresponding author upon reasonable request.
