# Supplementary material for: Exploring GBA1 gene in Parkinson's disease: Prevalence and variant spectrum from Asia minor
Source: Neurol Sci. 2025 Jun 20;46(9):4361–73. doi: 10.1007/s10072-025-08286-5 (PMC12394313; doi:10.1007/s10072-025-08286-5)
Supplement: Supplementary file 2 — Supplementary file2 (DOCX 17 KB) [file 10072_2025_8286_MOESM2_ESM.docx]

Supplementary table: Co-occuring genetic variants and clinical findings.

|  | *GBA1 + FBXO7* | *GBA1 + ADH1C* | *GBA1 +*  *PRKN* | *GBA1 + VPS13C* | *GBA1 + DJ-1* | *GBA1 + VPS35* |
| --- | --- | --- | --- | --- | --- | --- |
| Age (years) | 68 | 73 | 39 | 67 | 64 | 60 |
| Sex | Male | Female | Female | Male | Female | Female |
| Disease duration (years) | 4 | 5 | 0.5 | 12 | 20 | 9 |
| Symptoms duration (years) | 4 | 5 | 5 | 12 | 22 | 9 |
| LEDD (mg) | 375 | 570 | -* | 1690 | 1092 | 1150 |
| MDS-UPDRS-I | 3 | 9 | 0 | 12 | 22 | 3 |
| MDS-UPDRS-II | 20 | 15 | 1 | 26 | 32 | 10 |
| MDS-UPDRS-III | 47 | 50 | 37 | 49 | 40 | 27 |
| MDS-UPDRS-IV | 0 | 1 | 0 | 10 | 0 | 3 |
| MMSE | 23 | 26 | 29 | 29 | 19 | 29 |

MDS-UPDRS, Movement Disorders Soiety Unified Parkinson Disease Rating Scale; LEDD, Levodopa equivalent daily dose, MMSE, Mini-mental State Examination.

*De novo patient
